# Sensitive Detection of Rosmarinic Acid Using Peptide-Modified Graphene Oxide Screen-Printed Carbon Electrode

**DOI:** 10.3390/nano12193292

**Published:** 2022-09-22

**Authors:** Irina Georgiana Munteanu, Vasile Robert Grădinaru, Constantin Apetrei

**Affiliations:** 1 Department of Chemistry, Physics and Environment, Faculty of Sciences and Environment, “Dunărea de Jos” University of Galaţi, 47 Domneasca Street, 800008 Galaţi, Romania; 2 Faculty of Chemistry, Alexandru Ioan Cuza University, 11 Carol I Bd., 700506 Iasi, Romania

**Keywords:** rosmarinic acid, sensor, peptide, graphene oxide, cyclic voltammetry

## Abstract

Peptides have been used as components in biological analysis and fabrication of novel sensors due to several reasons, including well-known synthesis protocols, diverse structures, and acting as highly selective substrates for enzymes. Bio-conjugation strategies can provide a simple and efficient way to convert peptide-analyte interaction information into a measurable signal, which can be further used for the manufacture of new peptide-based biosensors. This paper describes the sensitive properties of a peptide-modified graphene oxide screen-printed carbon electrode for accurate and sensitive detection of a natural polyphenol antioxidant compound, namely rosmarinic acid. Glutaraldehyde was chosen as the cross-linking agent because it is able to bind nonspecifically to the peptide. We demonstrated that the strong interaction between the immobilized peptide on the surface of the sensor and rosmarinic acid favors the addition of rosmarinic acid on the surface of the electrode, leading to an efficient preconcentration that determines a high sensitivity of the sensor for the detection of rosmarinic acid. The experimental conditions were optimized using different pH values and different amounts of peptide to modify the sensor surface, so that its analytical performances were optimal for rosmarinic acid detection. By using cyclic voltammetry (CV) as a detection method, a very low detection limit (0.0966 μM) and a vast linearity domain, ranging from 0.1 µM to 3.20 µM, were obtained. The novelty of this work is the development of a novel peptide-based sensor with improved performance characteristics for the quantification of rosmarinic acid in cosmetic products of complex composition. The FTIR method was used to validate the voltammetric method results.

## 1. Introduction

In recent years, a remarkable area of study using nanotechnology has been the development and characterization of new functional and nutraceutical products from natural sources, in order to improve the quality of food without the need for the use of synthetic additives, and the design of new functionalities, especially related to health promotion, such as antioxidant, antimicrobial, and antitumor properties of food [1]. There is a growing concern for and continuous and advanced research on the use of polyphenolic compounds in both the food and pharmaceutical industries, as these compounds are considered supplements with preventive and curative properties for certain diseases [2].

**Figure 1:**
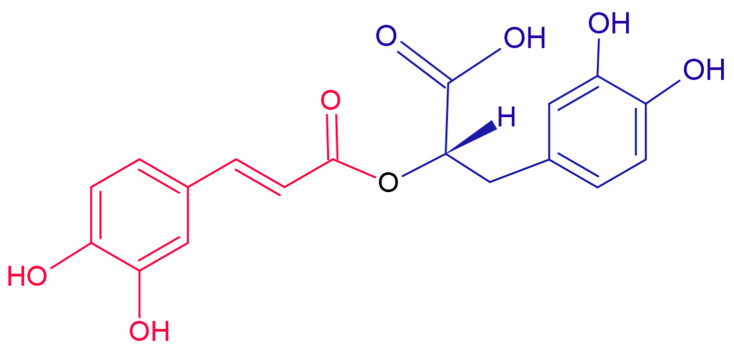
Chemical structure of rosmarinic acid.

Among polyphenolic compounds, rosmarinic acid has attracted considerable interest due to its important therapeutic properties and health benefits. The compound was first isolated by Scarpati and Oriente in 1958 [3] and is found in the composition of several medicinal plants of the Laminaceae family, including rosemary (*Rosmarinus officinalis*), mint (*Mentha spicata*), sage (*Salvia officinalis*), Spanish sage (*Salvia lavandulifolia*), marjoram (*Origanum majorana*), lemon balm (*Melissa officinalis*), basil (*Ocimum tenuiflorum*), oregano (*Origanum vulgare*), and thyme (*Thymus vulgaris*) [4,5,6]. Chemically, this compound is an ester of caffeic acid and 3,4-dihydroxyphenyl lactic acid (Figure 1) [7], containing in its structure two catechol moieties, thus having two pairs of ortho-hydroxyl groups grafted onto two aromatic rings [8], which are easily oxidizable and responsible for the electroactivity of this compound [9]. Rosmarinic acid has a remarkable medicinal value for the human body through its antioxidant [10], anti-inflammatory [11], anti-tumor [12], immunomodulatory [13], and also antimicrobial activity [14]. At the same time, the compound attenuates T lymphocyte receptors, with the effect of limiting allergic diseases such as conjunctivitis, rhinitis, or allergic asthma, but it is also a protector against neurotoxicity and can delay the progression of Alzheimer’s disease [15]. 

Rosmarinic acid, a naturally occurring compound along with other polyphenols, is difficult to evaluate and -determine from a complex system, due to interference phenomena, implying stringent requirements and conditions for the analytical methods used for this purpose [16]. Compared to existing approaches such as high-performance liquid chromatography [17], UV–Vis spectroscopy [18], ultra-fast liquid chromatography [19], and Fourier transform infrared spectroscopy (FTIR) [20], the simplicity of biosensors favors their use, with combining electroanalytical methods with the sensitivity of enzymatic reactions being one of the most encouraging methods for developing this type of device [21,22]. A series of biosensors using redox enzymes such as peroxidase, laccase, or tyrosinase have been developed in order to evaluate the total content of phenolic compounds expressed as rosmarinic acid equivalents in various samples such as pharmaceutical formulations, olive oil, or different natural extracts from plants [23,24,25]. Therefore, the development of new strategies for the construction of sensors and improvement of detection capacity is of great significance for the analysis of rosmarinic acid [8]. Using graphene oxide and peptides is an alternative to the detection systems that have been developed so far. 

Graphene is a unique two-dimensional material with spectacular structural and electronic properties that has appealed during the emergence of a new era of sensors. With focus on achieving improved performances, researchers are exploring the spectacular properties of graphene, such as its high specific surface area (theoretically, 2.630 m^2^/g for graphene with a single layer of carbon atoms) [26], outstanding electronic properties [27], strong mechanical resistance [28], and very good thermal and electrical conductivity [29], for applications in diverse fields [30]. A functionalized graphene-based material is graphene oxide (GO), which is one of the source materials for synthesis of graphene by chemical or thermal reduction processes [31]. Compared with the high cost of graphene film, GO is an easy-to-make material that has a similar structure. Both of these compounds have a hexagonal carbon lattice, but the GO sheet is usually distorted where it is bonded to the oxygen groups [32]. The main limitation for GO’s application is its low reproducibility, since a number of oxidized side chains are difficult to remove [33]. In recent years, the study of GO-based materials has garnered increasing interest due to GO’s simple synthesis, high dispersibility in a range of solvents, ability to couple electroactive species to the surface, and unique optical properties for cellular imaging and drug delivery [34,35]. Unlike conventional graphene, it provides a wide range of chemical methods for attachment of various functional groups (carbonyl, carboxyl, hydroxyl) to its surface for control of optical transparency and electrical and thermal conductance [36,37,38]. 

The literature from the last decade describes the use of peptides in state-of-the-art approaches for sensors with exhaustive applications [39]. Specific peptides are formed by natural or synthetic short polymers of amino acids, which are linked via peptide bonds with shorter lengths than those of proteins [40]. The main advantages of using these compounds are represented by their facile synthesis, stability, and selectivity towards a particular target compound. Peptides are versatile molecules that can self-assemble in different nanostructures controlled by non-covalent bonds such as electrostatic interactions, hydrogen bonds, and van der Waals interactions [41]. They can be modified with specific functional groups, thus making them suitable for the development of new biosensing platforms [42]. In addition, peptides are characterized by a high affinity for organic/inorganic compounds, a well-known structure, chemical stability, biocompatibility, and low immunogenicity, and do not imply complicated protocols or high synthesis costs [43]. These are good premises for their successful integration in the development of sensing devices for medical purposes. Furthermore, peptides are characterized as materials that prevent surface contamination because they contain natural amino acids that are biocompatible zwitterionic molecules and have hydrogen donor/acceptor properties due to the carbonyl, amino, and hydroxyl groups in the peptide structure [44]. The effect of non-specific adsorption on sensitive surfaces contributes to a major weakening of sensor function, limiting their practical application [45]. To date, various materials have been used to prevent surface contamination, such as silver ions [46], quaternary ammonium salts [47], hydrophilic polymers [48], zwitterionic materials [49], silica membrane [50], and poly (ethylene glycol) (PEG) [51]. Compared to the above materials, zwitterionic peptides are gradually becoming more widely studied. They can be rapidly synthesized and possess carboxyl (–COOH), amino (–NH_2_), and particularly amide (C(=O)NH-) groups, which can form a hydration layer on the surface via hydrogen bonds [52]. Due to hydration, peptides can resist the non-specific adsorption of proteins onto the detection surface. Additionally, the immobilized peptides and their quantifiable ligands may have similar structural moieties that could enhance their interaction.

**Figure 2:**
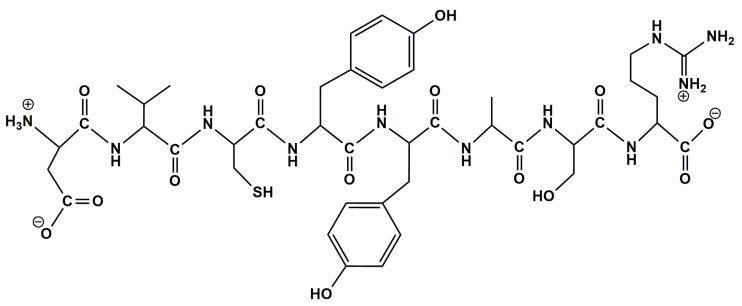
Chemical structure of peptide under study.

Therefore, the aim of this study is to fabricate a novel sensor, using a zwitterionic peptide (having a primary sequence H_2_N-D-V-C-Y-Y-A-S-R-COOH) (Figure 2), attached via a cross-linking agent to the surface of the screen-printed carbon electrode (SPCE) modified with a GO composite film, with applicability for the determination and quantification of rosmarinic acid in cosmetic products. Parameter optimization and sensor characterization were performed using CV as the electrochemical method of choice. Currently, there are no studies on the detection of rosmarinic acid with a GO and peptide-modified screen-printed sensor, and future research directions should consider the application of this device with other types of samples, such as food products, nutraceuticals, or biological samples such as human serum.

## 2. Materials and Methods

### 2.1. Reagents and Solutions

To obtain the novel sensor, GO-modified SPCE (SPCE/GO), purchased from Metrohm DropSens (Oviedo, Spain), was used and subsequently modified in the laboratory. This modification consisted of adding peptide to the sensor surface followed by cross-linking, thus obtaining the GO-Peptide/SPCE sensor.

Octapeptide H_2_N-D-V-C-Y-Y-A-S-R-COOH, where D is aspartic acid, V is valine, C is cysteine, Y is tyrosine, A is alanine, S is serine, and R is arginine, was purchased from ProteoGenix, Schiltigheim, France. Peptide purity >95% was confirmed by RP-HPLC chromatography on a Dionex UltiMate 3000 UHPLC system (Thermo Scientific, Waltham, MA, USA) using a mobile phase of 0.1% TFA (trifluoroacetic acid, Sigma-Aldrich, Steinheim, Germany) in a gradient of ACN (Acetonitrile, HPLC grade, Merck, Germany). The retention time was identical with the elution time observed initially for a previous peptide, obtained by solid-phase synthesis strategy, used as a standard compound (unpublished data).

To immobilize the peptide on the sensor surface, a solution of 10 mg/mL peptide in 0.1 M phosphate buffer solution (PBS) (pH = 6.5) was used.

The following reagents used were purchased from Sigma-Aldrich (St. Louis, MO, USA) and were used without further purification: potassium chloride (KCl), potassium ferrocyanide (K_4_[Fe(CN)_6_]), potassium ferricyanide (K_3_[Fe(CN)_6_]), sodium diphosphate (NaH_2_PO_4_), and phosphoric acid (H_3_PO_4_). K_4_[Fe(CN)_6_] 1 mM and PBS 0.1 M solutions were used in preliminary experiments.

NaH_2_PO_4_ and H_3_PO_4_ were used to prepare PBS 0.1 M, the amount of NaH_2_PO_4_ being calculated, weighed, and dissolved in ultrapure water (18.3 MΩ · cm, Milli—Q Simplicity^®^ Water Purification System from Millipore Corporation) (Bedford, MA, USA). Adjusting the pH to values between 4.0 and 8.0 (4.0, 4.5, 5.0, 5.5, 6.0, 6.5, 7.0, 7.5, 8.0) was carried out by adding 0.1 M H_3_PO_4_ or NaOH 2 M, with subsequent pH measurement using a pH meter from WTW instruments, Weilheim, Germany. 

Rosmarinic acid was purchased from Sigma-Aldrich and was of analytical purity. A 10^−3^ M rosmarinic acid stock solution was prepared by dissolving the appropriate amount of rosmarinic acid in PBS, pH = 6.5.

### 2.2. Electrodes and Devices Used

The electrochemical cell used had a capacity of 50 mL, and the electrode system was formed from counter electrode, which was platinum wire, and reference electrode: Ag/AgCl. The working electrode was the modified electrode with peptide (GO-Peptide/SPCE).

Cyclic voltammetric measurements were performed in an EG&G PARC Model 263 potentiostat/galvanostat (Princeton Applied Research Corp., Oak Ridge, TN, USA) connected to a desktop computer and controlled by software (Echem). The Partner AS 220/C/2 analytical balance was used for weighing compounds, and the Elmasonic ultrasonic bath (Carl Roth GmbH, Karlsruhe, Germany) was used for dissolution homogenization. 

The FTIR spectra were obtained with a Bruker ALPHA FT-IR (BrukerOptik GmbH, Ettlingen, Germany) spectrometer, which uses OPUS (BrukerOptik GmbH, Ettlingen Germany) software in the range of 4000–500 cm^−1^ (32 scans, 4 cm^−1^ resolution) in the total attenuated reflected mode (ATR). Between the measurements, the ZnSe crystal was cleaned with ultrapure water and isopropanol. The spectra were recorded towards the air as background.

The surface morphology of the samples was examined with a Verios G4 UC scanning electron microscope (Thermo Scientific, Waltham, MA, USA) equipped with an energy-dispersive X-ray spectroscopy analyzer (Octane Elect Super SDD detector (AMETEK, Tokyo, Japan). The samples were coated with 10 nm platinum using a Leica EM ACE 200 Sputter Coater (Leica Microsystems, Vienna, Austria) to provide electrical conductivity. Then, the samples were fixed with double adhesive tape on cylindrical Al conducting supports. SEM investigations were performed in High Vacuum mode using a secondary electron detector (Everhart–Thornley detector, ETD) at an accelerating voltage of 5 kV.

Fluorescence (emission) spectra were recorded using an FP-8350 Spectrofluorometer (JASCO, Tokyo, Japan). Spectrofluorimetric titrations were performed by excitation of peptide solution, initially dissolved in DMSO and diluted in 0.1 M PBS, pH 6.5, using an excitation length of 275 nm, with a scanning step of 0.5 nm, scanning rate of 1000 nm/min, and a number of 5 acquisitions for each emission spectrum. Both excitation and emission slits were selected at 5 nm. Octa-peptide emission spectra were recorded in the range 285–500 nm. The 3D fluorescence spectra of the peptide in the absence or presence of ferricyanide were recorded with a scan rate of 2000 nm/min, scan pitch of 1 nm, excitation range 200–369 nm, and emission range 210–380 nm.

### 2.3. Sensor Preparation

**Figure 3:**
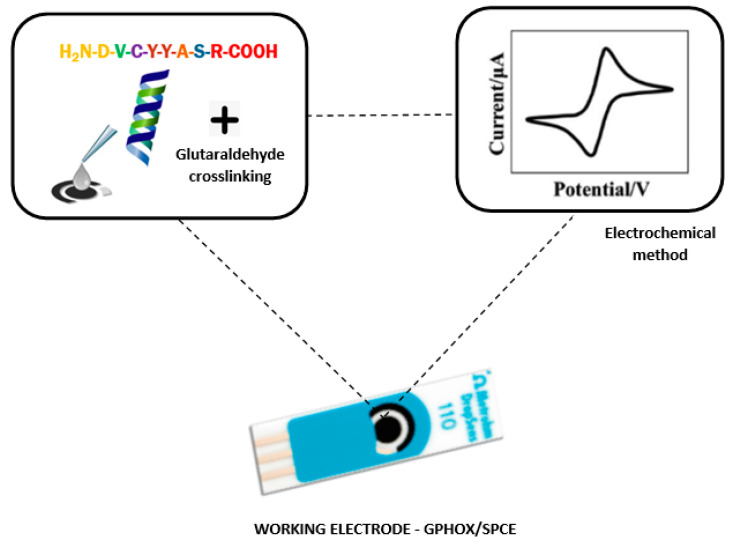
Preparation process of GO-Peptide/SPCE sensor.

The GO/SPCE electrode was used as support to prepare the sensor (Figure 3). Briefly, a stock solution of peptide was prepared, and 20 μL of this solution was drop-casted onto the working electrode, sequentially, in four steps (5 µL in each step) and then allowed to dry for about two hours between each step. Glutaraldehyde 2% (*v*/*v*) was used as an immobilisation agent.

The resulting sensor was stored at 4 °C until use, for a period not exceeding 72 h [53].

### 2.4. Methods of Analysis

CV was used to characterize working electrodes as well as the stage of rosmarinic acid detection in the solution prepared with pure substance and in the solutions prepared with the cosmetic samples.

CV is a powerful and common electrochemical technique commonly employed to investigate the reduction and oxidation processes of molecular species. CV is also invaluable to study electron-transfer-initiated chemical reactions, which includes catalysis. With this technique, the potential of the cell is changed with a given rate between chosen potential limits, and the current response is followed [54,55]. 

### 2.5. Samples and Preparation of Solutions to Be Analyzed

The cosmetics included in the analysis were purchased from health food stores, with the pharmaceutical form of presentation for all three products being cream. The products contain a series of active ingredients and auxiliary compounds, the rosemary leaf extract content being indicated on the label of each one. 

For the electrochemical analysis, 3 different amounts of each product were used and mixed with 50 mL 0.1 M PBS of pH 6.5, resulting in homogeneous solutions. Therefore, three samples were prepared from each of the three cosmetics and analyzed separately (in triplicates for each cosmetic product). 

## 3. Results and Discussion

### 3.1. Electrode Characterization

The construction of a versatile peptide-based sensor entails obtaining analytically important features such as sensitivity, linearity, response time, reliability, and accuracy, each of which is directly related to peptide binding to the electrode surface [56]. 

In order to highlight the changes in the commercial GO-based sensor, its active surface was analyzed using two methods: on the one hand, FTIR spectrometric method, and on the other hand, scanning electron microscopy (SEM). 

#### 3.1.1. FTIR Spectrometric Method

**Figure 4:**
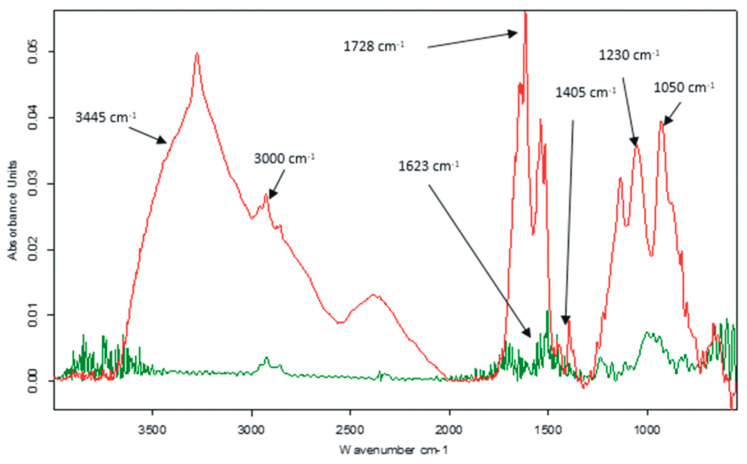
FTIR spectra for GO/SPCE (green line) and GO-Peptide/SPCE (red line).

The first method used to characterize the modified sensor was the infrared spectrometric method. The results are shown in Figure 4. 

Several peaks representative of the presence of GO can be noticed in the wavenumber range 1050–1728 cm^−1^ [57]. The absorption at about 1623 cm^−1^ is associated with the stretching vibration of the benzene ring C=C [58]. At the same time, the band at 1728 cm^−1^ occurs due to the C=O [59], while the band at 1405 cm^−1^ can be attributed to the C–O [60], which includes both the 1230 cm^−1^ epoxy C–O [61] and the 1050 cm^−1^ alkoxy C–O [62], located at the edges of the graphene oxide.

Peptide-sequence-related peaks can be easily noted. Thus, in the range of 1600–1700 cm^−1^, type I amide bands are found [63]. This interval includes the C=O stretching vibrations of the peptide sequence and usually has a broad contour, composed of several overlapping bands due to different polypeptide segments with different secondary structures [64]. Furthermore, stretching vibrations corresponding to free or non-hydrogen bonding -NH_2_ groups occur at 3445 cm^−1^ and 3438 cm^−1^, respectively [65], while the absorption corresponding to the wavenumber 3000 cm^−1^ and 3250 cm^−1^ is attributed to the O-H stretching vibration of the hydroxyl groups in the serine and tyrosine structure [66].

#### 3.1.2. Morphological Characterization Using SEM

**Figure 5:**
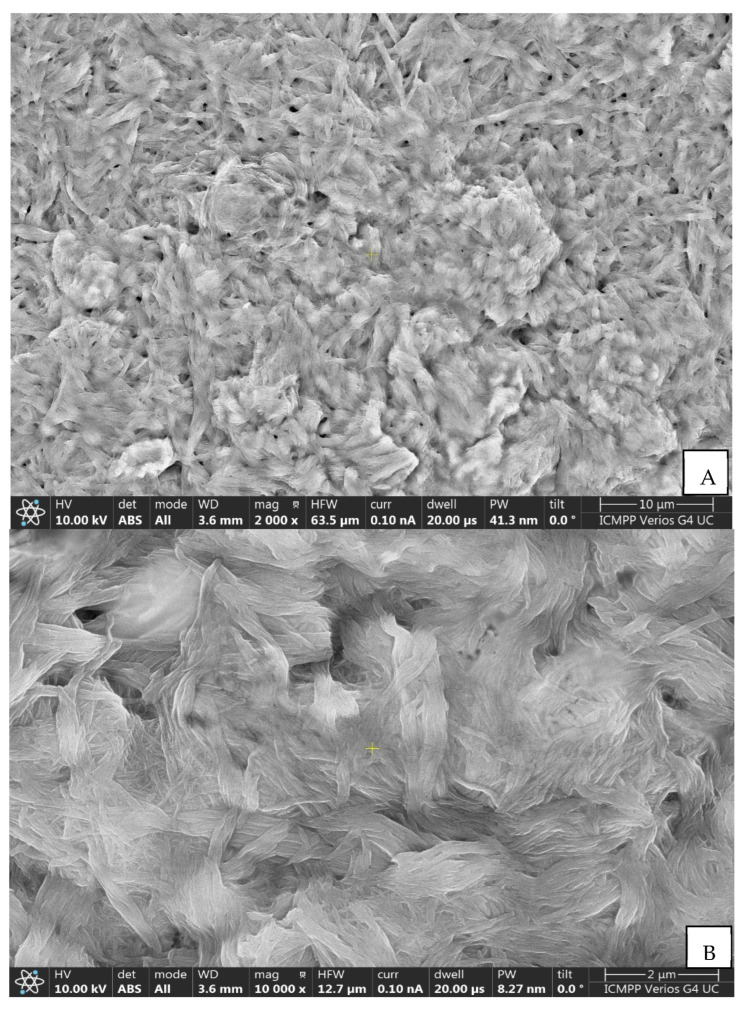

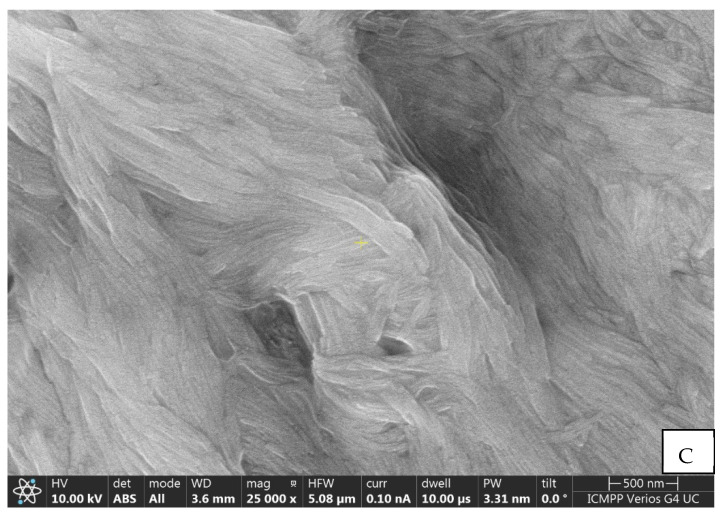
Scanning electron micrograph at different magnifications representing the active surface of GO-Peptide/SPCE: (**A**) 2000 times magnification; (**B**) 10,000 times magnification; (**C**) 25,000 times magnification.

Figure 5 depicts the SEM images, emphasizing the surface morphology of the composite nanofilm containing GO and peptide. 

At the first magnification (Figure 5A), the fibers arranged as self-assembled microfluidic channels are observed [67]. At 10,000 times magnification (Figure 5B), it is noticed that the fibrils are wavy and interwoven [68], and at 25,000 times magnification (Figure 5C), some fibers appear to have a twisted, rope-like structure [69]. In general, the morphological diversity of peptides and their ability to self-assemble can be elucidated and demonstrated on the basis of the hydrophobicity of the constituent amino acids of the peptide sequence and also on the basis of the polarity of the solvent [70].

### 3.2. Optimization of Experimental Parameters

Initially, two experimental parameters were studied and optimized to achieve the best performance of the peptide-based sensor, namely the pH and the amount of peptide used to modify the GO/SPCE sensor. The response of the GO-Peptide/SPCE was based on the anodic peak current obtained in 0.1 M PBS, using CV. 

#### 3.2.1. Influence of pH

To decide the pH value at which further determinations should be made in this study, the electrochemical behavior of the sensor was evaluated in 0.1 M PBS with different pH values (4.0, 4.5, 5.0, 5.5, 6.0, 6.5, 7.0, 7.5, 8.0) at a scan rate of 0.05 V·s^−1^ [54,71]. According to previous research, it was established that the optimum pH value for the detection of phenolic compounds is between 6.0 and 7.0. Peaks obtained in this pH range are more obvious and well defined. In addition, a lower pH value could contribute to a faster degradation of the peptide [72].

Upon immersion of GO-Peptide/SPCE in 0.1 M PBS at different pH values, cyclic voltammograms showed, in all cases, two peaks: a more obvious anodic one and a cathodic one of low intensity. It can be clearly seen that for pH higher than 7.0, the response decreases significantly, and a maximum response is reached at pH values around 6.5. 

**Figure 6:**
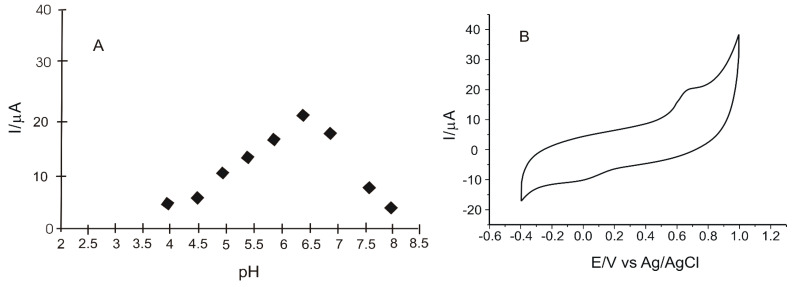
Influence of pH on the intensity of the anodic peak current (**A**). Cyclic voltammogram of GO-Peptide/SPCE immersed in 0.1 M PBS, pH = 6.5 (**B**).

At this pH value, the anodic peak occurs at 0.67 V and the cathodic peak occurs at −0.01 V. The appearance of these peaks is related to the electrochemical process of irreversible oxidation reduction of the peptide on the surface of the peptide-based sensor. Figure 6 shows the influence of pH on the peptide reduction process on the electrode surface (Figure 6A) and the cyclic voltammogram of GO-Peptide/SPCE immersed in 0.1 M PBS solution, pH = 6.5 (Figure 6B).

#### 3.2.2. Influence of the Amount of Peptide

**Figure 7:**
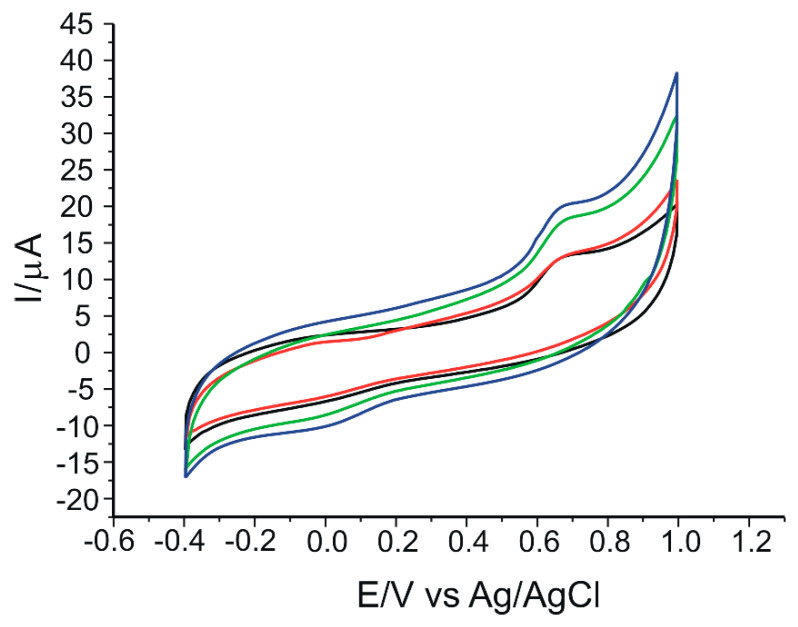
Cyclic voltammograms of sensors modified with: 10 µL peptide solution, uncross-linked (black line); 10 µL peptide solution, cross-linked (red line); 20 µL peptide solution, uncross-linked (green line); 20 µL peptide solution, cross-linked (blue line). All sensors immersed in 0.1 M PBS, pH = 6.5. Scan rate: 0.05 V·s^−1^.

To determine the optimal amount of peptide solution required to modify the sensor, two different volumes of this solution, 10 µL and 20 µL, were used and added sequentially in several steps (5 µL at each step) to the sensor surface. Furthermore, to study the influence of cross-linking on peptide immobilization, the two sensors containing 10 µL and 20 µL peptide solution were cross-linked, while two uncross-linked sensors containing the same volumes of peptide solution were used in parallel. Cyclic voltammograms recorded for all these sensors immersed in 0.1 M PBS pH = 6.5 at a scan rate of 0.05 V·s^−1^ are shown in Figure 7. It is obvious that for the sensor modified with 20 µL peptide solution and cross-linked, the anodic peak current is more intense and better defined (19.88 µA). Therefore, for the following determinations of this study, the cross-linked sensor modified with 20 µL peptide solution was used.

### 3.3. Electrochemical Properties of GO-Peptide/SPCE in K_4_[Fe(CN)_6_]/K_3_[Fe(CN)_6_] Solution

The next step consisted in analyzing the electrochemical response of both the modified and unmodified sensor in a solution containing 10^−3^ M K_4_[Fe(CN)_6_]/K_3_[Fe(CN)_6_] dissolved in 0.1 M PBS, pH = 6.5, recording cyclic voltammograms from −0.4 to 1.0 V. 

**Figure 8:**
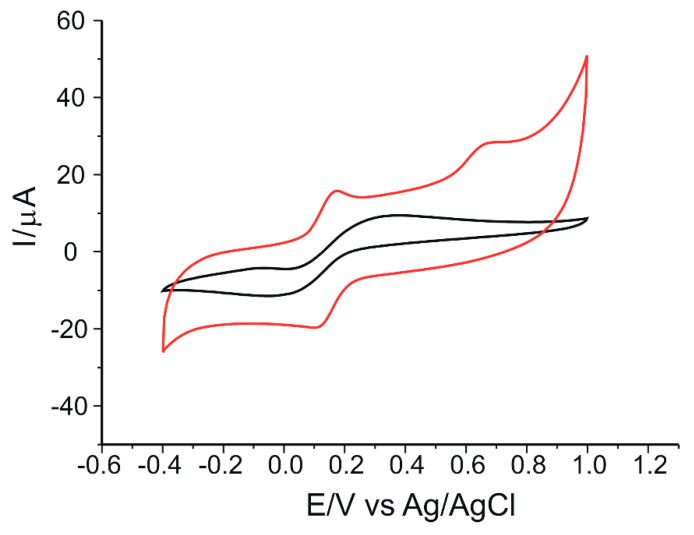
Cyclic voltammograms of GO/SPCE (black line) and GO-Peptide/SPCE (red line) immersed in 10^−3^ M K_4_[Fe(CN)_6_]/K_3_[Fe(CN)_6_]-0.1 M PBS solution, at the scan rate of 0.05 V·s^−1^.

Figure 8 describes the stable sensor signals. It can be seen that, for the GO/SPCE, there is a pair of redox peaks, one anodic and one cathodic. For the modified sensor, two anodic peaks of different intensities and potentials and one cathodic peak are observed. The pair of peaks found for both sensors is due to the reversible oxidation of the ferrocyanide ion to ferricyanide, which occurs at the surface of the electrodes. The second oxidation peak in GO-Peptide/SPCE is more intense than the first and can be attributed to the formation of the peptide film on the electrode surface. 

As can be seen from the table, both sensors show a cathodic peak current/anodic peak current (Ipc/Ipa) ratio that exceeds the ideal value of 1. This demonstrates that the redox process is quasi-reversible [73]. However, the peptide-modified electrode has a higher degree of reversibility, the separation between anodic peak potential (Epa) and cathodic peak potential (Epc) (ΔEp) being three times smaller compared to GO/SPCE, and the value is closer to the ideal value. 

It is noted that Ipa and Ipc are higher for the GO-Peptide/SPCE sensor (Ipa = 15.58 µA and Ipc = −19.42 µA) compared to the values obtained with the unmodified electrode. Considering these results, the highest sensitivity for ferrocyanide ion detection was achieved with GO-Peptide/SPCE. At the same time, the modification of the sensor with peptide contributed to increasing the electron transfer rate on the surface of the sensor by improving its reversibility [16]. 

Both electrodes show similar electrochemical responses in accordance with the parameters obtained and can be successfully applied in further analysis. 

**Figure 9:**
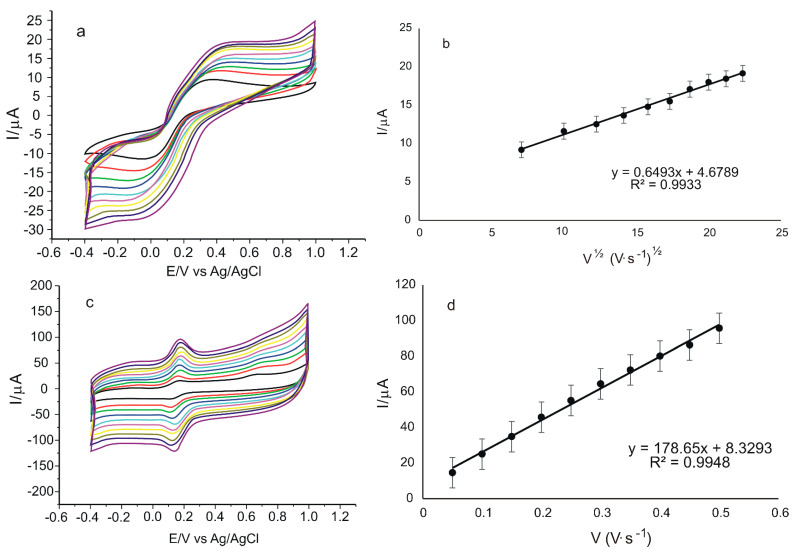
Cyclic voltammograms of GO/SPCE (**a**) and GO-Peptide/SPCE (**c**) immersed in 10^−3^ M K_4_[Fe(CN)_6_]/K_3_[Fe(CN)_6_]-0.1 M PBS solution at pH = 6.5 recorded at scan rates between 0.05 and 0.5 V·s^−1^. Linear dependence of Ipa and square root of scan rate in the case of GO/SPCE (**b**) and linear dependence of Ipa and scan rate in the case of GO-Peptide/SPCE (**d**).

To study the kinetics of the redox process, cyclic voltammograms were recorded for GO/SPCE and GO-Peptide/SPCE using 10^−3^ M K_4_[Fe(CN)_6_]/K_3_[Fe(CN)_6_]-0.1 M PBS solution at pH = 6.5 with scan rates ranging from 0.05 to 0.5 V·s^−1^. The results obtained are presented in Figure 9. 

Figure 9a,c show that the current increases with the increasing scan rate. In order to check the determining factor of the oxide-reduction process, linear regression models were described, correlating Ipa with the scan rate or the square root of the scan rate.

For GO/SPCE, a linear dependence is observed between Ipa and the square root of the scan rate, as depicted in Figure 9b. This linear dependence suggests that the oxidation reduction of potassium ferrocyanide is controlled by the diffusion of the electroactive species, this being the determining stage of the electrochemical process [74]. In the case of GO-Peptide/SPCE, there is a linear dependence between Ipa and scan rate, which suggests that the electrochemical process at the electrode surface is controlled by the adsorption of the electroactive species [75] (Figure 9d). This change in the determining factor of the oxide-reduction process in the case of GO-Peptide/SPCE is due to the modification of the sensor with the peptide, which has a major effect on the reaction kinetics at the electrode surface and provides a suitable environment for a sensitive layer–analyte interaction and rapid electron transfer [76]. 

Fluorescence quenching experiments on the peptide with ferricyanide or ferrocyanide were also performed. It was found that, at low concentrations, ferricyanide ions have a higher quenching capacity than ferrocyanide ions. Although the dynamic quenching constant of ferricyanide ions was about 40% higher than that of ferricyanide ions, the static quenching was more pronounced for the latter. 

Furthermore, 3D spectra of octapeptide have been registered both in the absence and in the presence of ferricyanide ions. Regarding peptide, two signals were observed at the ex/em pairs of 235/302 and 275/303, respectively. In the presence of ferricyanide ions (5 equivalents), there was a slight shift in the emission maximum (at 304 nm) at the excitation length of 235 nm and a decrease in the emission signals by about 40%. This phenomenon may suggest interaction between the ferricyanide ions and the phenolic nuclei of the peptide. A similar behavior has been reported for human transferrin, in which the interaction of the protein with iron ions is mediated by two tyrosine residues [77]. Another Raman spectroscopy study suggests that a tyrosine residue and carboxylate groups of glutamate and aspartate from amyloid β peptide might be involved in binding of Fe(III) [78]. In particular, our peptide possesses two carboxyl groups situated at extremities and two phenol functional groups in the central core. 

### 3.4. Electrochemical Responses of Sensors in Rosmarinic Acid Solution

Electrochemical behavior of GO/SPCE and GO-Peptide/SPCE in rosmarinic acid solution was investigated using CV.

**Figure 10:**
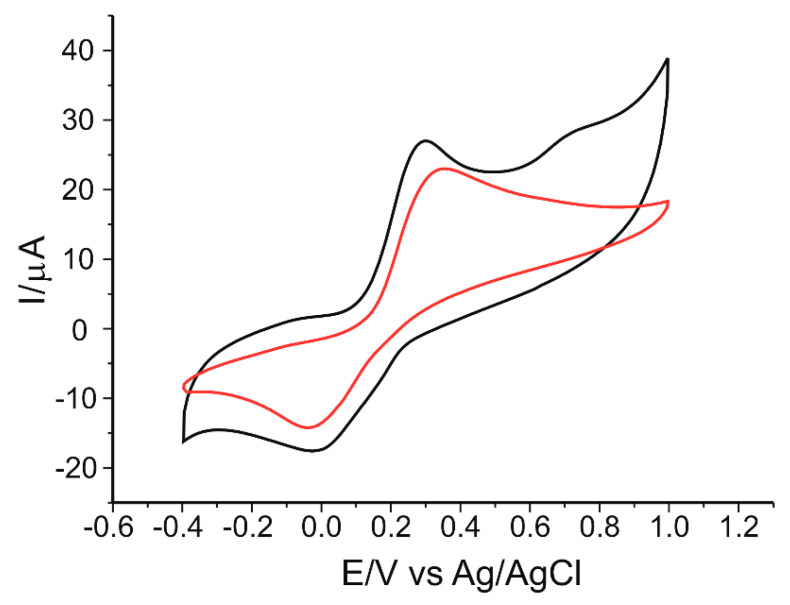
Cyclic voltammograms of GO/SPCE (red line) and GO-Peptide/SPCE (black line) immersed in 10^−3^ M rosmarinic acid-0.1 M PBS solution (pH 6.5). Scan rate: 0.05 V·s^−1^.

Figure 10 shows the response of the two electrodes, GO/SPCE and GO-Peptide/SPCE, when immersed in a solution of 10^−3^ M rosmarinic acid-0.1 M PBS (pH = 6.5). Three cycles in the optimized potential range (−0.4 V to 1.0 V) were required to achieve a stable sensor response. The cyclic voltammograms shown in Figure 10 were achieved after stabilizing the signals.

In both cases, a pair of well-defined peaks having different intensities is shown, namely an anodic peak, representing the oxidation of rosmarinic acid to the corresponding o-quinone, and a cathodic peak, corresponding to the electrochemical reduction back to rosmarinic acid. In the case of the peptide-modified sensor, a shift in the oxidation potential towards more negative values and an increase in Ipa was observed, demonstrating that the peptide, in combination with GO, confers an improved conductivity to the sensor and a thermodynamically favored antioxidant activity to the compound [79]. In the case of GO-Peptide/SPCE, the appearance of the second anodic peak at the potential value of 0.72 V due to the presence of peptide on the SPCE surface is observed, as previously demonstrated in the experimental parameter optimization studies. Hydrogen bonds between the amide and/or phenolic groups in the peptide structure and the phenolic and/or carboxyl groups of rosmarinic acid could contribute to improving the interaction with GO, thereby favoring electron transfer [80]. 

The presence of the two tyrosine residues may confer antioxidant properties to this peptide, with the possibility of free radical neutralization [81]. Rosmarinic acid has been shown to interact with the structural motif ^306^VQIVYK^311^ of the tau protein and is thus involved in the anti-aggregation mechanism of β-amyloid peptide assembly [82]. Rosmarinic acid has a better efficiency for quenching tryptophan fluorescence in bovine serum albumin (BSA) compared to caffeic and salvianic acids [83]. 

**Figure 11:**
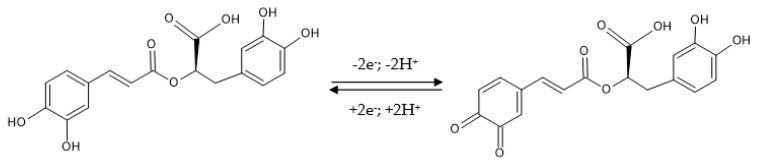
The oxidation mechanism of rosmarinic acid.

Although rosmarinic acid contains two catechol moieties, corresponding to caffeic acid and 3,4-dihydroxyphenyl lactic acid, respectively, its cyclic voltammogram shows a single anodic peak at a potential of 0.29 V, suggesting that the oxidation potentials of each catechol group are very close at pH 6.5, their peaks becoming indistinguishable [84]. Therefore, the electrochemical oxidation of rosmarinic acid follows a mechanism of catechol-type compounds, i.e., a two-electron and two-proton transfer process (Figure 11). 

GO-Peptide/SPCE stands out for a low Epa value, suggesting that the oxidation process involves a lower activation energy and is closely related to the presence of the peptide. Furthermore, taking into account that in the structure of rosmarinic acid the carboxylic group is not directly bound to the aromatic ring, the electron attraction effect will be supported by the presence of the double bond; the positive inductive effect of the alkyl chain increases the electron density on the aromatic ring, lowering the electrochemical oxidation potential [85]. In the literature, higher oxidation peak values have been reported for detection of rosmarinic acid using the glassy carbon electrode (GCE) at 0.63 V [8] and carbon paste electrode (CPE) at 0.38 V [86]. The lower redox potential obtained for the oxidation peak corresponding to rosmarinic acid in this work is due to the use, for the construction of the new sensor, of SPCE modified with the GO composite film, on the surface of which the zwitterionic peptide was fixed.

The low value of Epa indicates a fast electron transfer process in the redox reaction of rosmarinic acid at the active surface of the modified sensor. Moreover, GO-Peptide/SPCE showed the highest degree of reversibility, with ΔEp value smaller than in the GO/SPCE sensor, and also with the Ipc/Ipa ratio higher in the peptide-modified sensor compared to the unmodified sensor. Taking this into account, we can state that GO-Peptide/SPCE shows the highest degree of reversibility. Furthermore, for this peptide-based sensor, the highest peak current values were obtained for both the oxidation and reduction processes of rosmarinic acid. Therefore, from the experimental values achieved, we can conclude that the highest sensitivity in the detection of rosmarinic acid was obtained for GO-Peptide/SPCE.

### 3.5. Kinetics of the Responses

The next stage was to study the electrochemical behavior of GO/SPCE and GO-Peptide/SPCE in 10^−3^ M rosmarinic acid-0.1 M PBS solution at pH 6.5, applying scan rates ranging from 0.05 to 0.5 V·s^−1^. Considering that the anodic peaks are higher and better defined, the dependence of Ipa on the scan rate or square root of the scan rate will be further analyzed.

**Figure 12:**
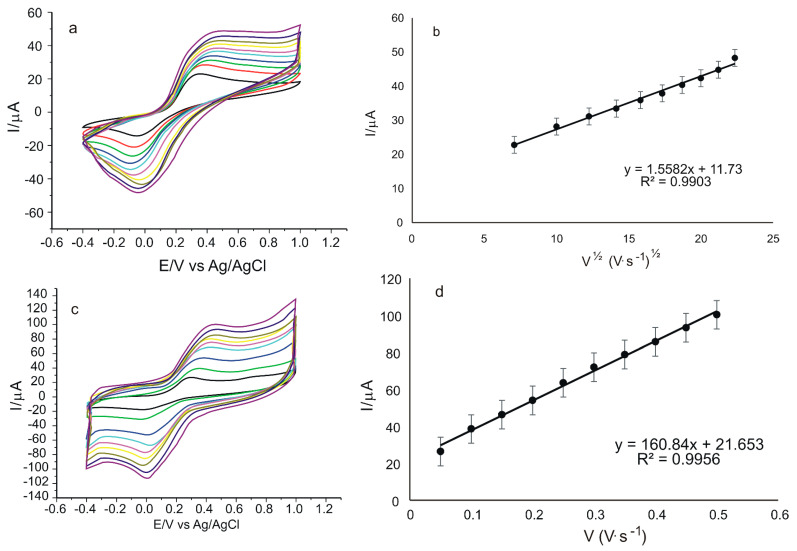
Cyclic voltammograms of GO/SPCE (**a**) and GO-Peptide/SPCE (**c**) immersed in 10^−3^ M rosmarinic acid-0.1 M PBS at pH = 6.5 recorded at scan rates between 0.05 and 0.5 V·s^−1^. Linear dependence between Ipa and square root of scan rate in the case of GO/SPCE (**b**) and linear dependence between Ipa and scan rate in the case of GO-Peptide/SPCE (**d**).

Figure 12 describes the cyclic voltammograms of the two electrodes, recorded at different scan rates.

From Figure 12a,c, a progressive increase in peak intensity can be seen with increasing scan rate. As in previous determinations in electroactive 10^−3^ M K_4_[Fe(CN)_6_]/K_3_[Fe(CN)_6_-0.1 M PBS solution, a linear dependence between Ipa and the square root of the scan rate is observed for GO/SPCE (Figure 12b), and a linear dependence between Ipa and scan rate is observed for GO-Peptide/SPCE (Figure 12d). Therefore, for GO/SPCE, the electrochemical process at the electrode surface is controlled by the diffusion of the electroactive species, and for GO-Peptide/SPCE, it is controlled by the adsorption of the electroactive species. This change in the kinetic determinant of the oxidation-reduction process at the electrode surface in the case of rosmarinic acid demonstrates, once again, the role and influence of the peptide sensor modification on the kinetics of the reaction at the electrode and implicitly on the electron transfer process.

### 3.6. Influence of Rosmarinic Acid Concentration on the Voltammetric Response of GO-Peptide/SPCE 

To determine the applicability of the sensor in practice, the influence of concentration on the peptide-based sensor response was evaluated. For this, cyclic voltammograms of GO-Peptide/SPCE in rosmarinic acid solution were recorded upon successive addition of varying amounts, between 5 µL and 1000 µL, of 10^−3^ M rosmarinic acid stock solution in 50 mL 0.1 M PBS at pH 6.5. Each volume addition step was followed by stirring. After mixing the test solution, cyclic voltammograms were registered. The concentration range studied was 0.1 µM–3.20 µM. The scan rate was 0.05 V·s^−1^, and the potential range was between −0.4 V and 1.0 V.

**Figure 13:**
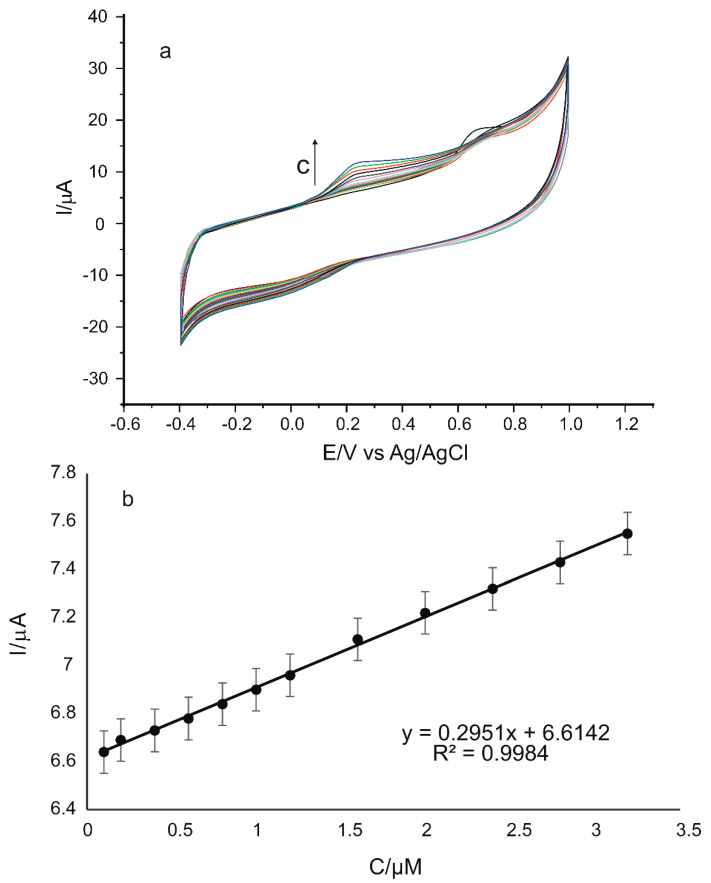
Cyclic voltammograms recorded for GO-Peptide/SPCE with the concentration between 0.1 and 3.20 µM rosmarinic acid (**a**); Linear dependence between Ipa and rosmarinic acid concentration in the range 0.1–3.20 µM (**b**).

The electrochemical responses of the sensor recorded by CV are shown in Figure 13a. It can be observed that the anodic and cathodic peak intensities increase with the increase in concentration.

In Figure 13b, the dependence between Ipa and the rosmarinic acid concentration in the range of 0.1–3.20 µM can be observed, in the case of using GO-Peptide/SPCE.

The plot illustrating the dependence between the Ipa values and the concentrations in the 0.1–3.20 μM range shows a linear dependence and a determination coefficient close to the ideal value 1 for our modified sensor. Therefore, limit of detection (LOD) and limit of quantification (LOQ) were calculated by using the calibration equations corresponding to the concentration range envisaged [87]. These two parameters were calculated using the equations LOD = 3σ/m and LOQ = 10σ/m, where σ is the standard deviation (SD) of the electrochemical signal for the control sample at the potential corresponding to the rosmarinic acid peak, and m is the slope of the calibration curve [88]. The results obtained are summarized in Table 1.

Due to the presence of the peptide, which favors the interaction with rosmarinic acid and gives the sensor sensitivity, it outperforms other sensors used to determine rosmarinic acid, as shown in Table 2. 

As the LOD and LOQ values obtained with our new GO-Peptide/SPCE for the detection of rosmarinic acid were higher than those in the other studies performed, as shown in Table 2, the analysis was continued on real samples, namely on cosmetic products.

### 3.7. Quantitative Determination of Rosmarinic Acid in Cosmetic Products

Taking into consideration its superior characteristics, the GO-Peptide/SPCE sensor was used in subsequent analysis to quantify rosmarinic acid in three cosmetic products, namely Apiterra anti-aging cream for sensitive skin, Sabio soothing and repairing balm with herbal extract, and Vivanatura Mattifying Moisturizing Cream for mixed-type skin.

All three products have beneficial properties, especially for sensitive skin, including anti-inflammatory, anti-irritant, astringent, repairing, moisturizing, antioxidant, and anti-ageing effects, providing the complexion with protection, intense hydration, and a toned and velvety look.

Two methods were used for the analysis of the three cosmetic products, namely CV (the method previously described in this work) and FTIR (Fourier transform infrared spectroscopy) (the standard method) [90]. The purpose of this analysis was, on the one hand, to demonstrate the feasibility of the voltammetric method for the quantitative analysis of rosmarinic acid, and, on the other hand, to compare the results obtained by these two methods.

**Figure 14:**
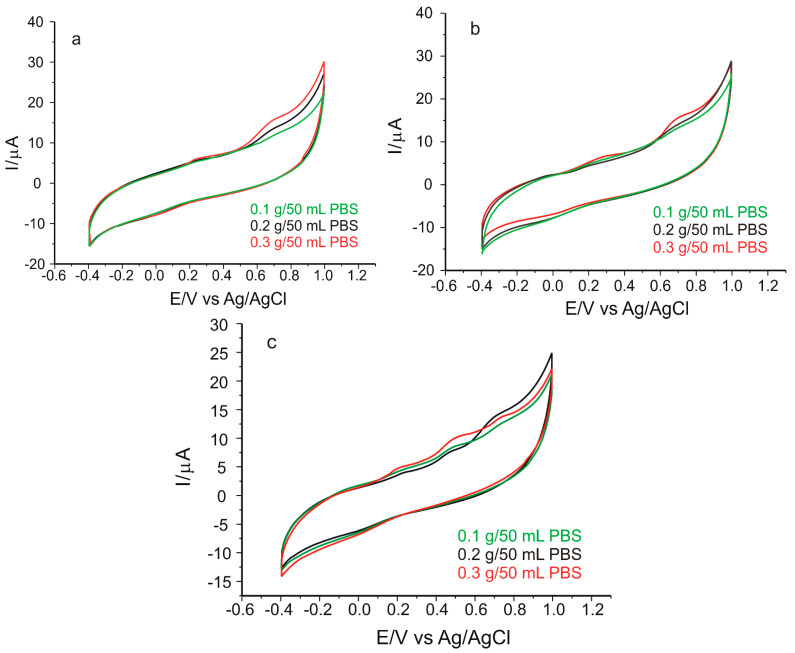
Cyclic voltammograms of GO-Peptide/SPCE immersed in solutions of (**a**) Apiterra anti-aging cream, (**b**) Sabio soothing and repairing balm, and (**c**) Vivanatura moisturizing mattifying cream of different concentrations. Scan rate: 0.05 V·s^−1^.

Three different well-established amounts of each product were used to obtain the solutions to be tested: 0.1 g, 0.2 g, and 0.3 g. Applying CV, measurements were recorded from −0.4 V to 1.0 V, at a scan rate of 0.05 V·s^−1^ (Figure 14).

In the case of all the voltammograms shown in Figure 14, the appearance of the anodic peak (at potentials of 0.238 V for (a), 0.227 V for (b), and 0.207 V for (c)) and the cathodic peak (at potentials of −0.033 V for (a), −0.021 V for (b), and −0.018 V for (c)) was related to the presence of rosmarinic acid in the samples (taking these values into account, rosmarinic acid has been quantitatively determined in the selected samples), and the anodic peak was also related to the presence of the peptide (at the potential value of 0.718 V for (a), 0.717 V for (b), and 0.708 for (c)). In Figure 14c, in addition to the above-mentioned peaks, an anodic peak at a potential value of 0.492 V is also shown, related to the presence of other compounds in the cosmetic product in question. 

On the basis of the anodic peak current, the amount of cosmetic product taken in the analysis, and the equation of the calibration line, the concentrations of rosmarinic acid in cosmetics were calculated, and the results are included in Table 3. All the analyses were carried out in triplicate. 

The second method used to determine rosmarinic acid from real samples and to verify the feasibility of the voltammetric method was the FTIR spectrometric method. The samples to be analyzed were prepared by mixing with potassium bromide, without other pre-treatment steps. A standard sample prepared from pure rosmarinic acid and potassium bromide was used, with the concentration of rosmarinic acid being 1%. All experiments were carried out in triplicate. The wave number corresponding to the C=O group was 1718 cm^−1^ [91]. Accordingly, the absorbance for the standard sample and the cosmetic samples was measured at this wavenumber. Taking into account the results obtained, the concentrations of rosmarinic acid in the three cosmetic products analyzed were calculated as shown in Table 3.

The values obtained by using the FTIR method are similar to those obtained by CV. Analysis of variance showed that there is no significant difference between the results obtained with both methods at a 99% confidence level, which demonstrates that the voltammetric method using the new GO-Peptide/SPCE sensor is useful for the quantification of rosmarinic acid with adequate precision and selectivity, this representing the present study’s novelty.

**Figure 15:**
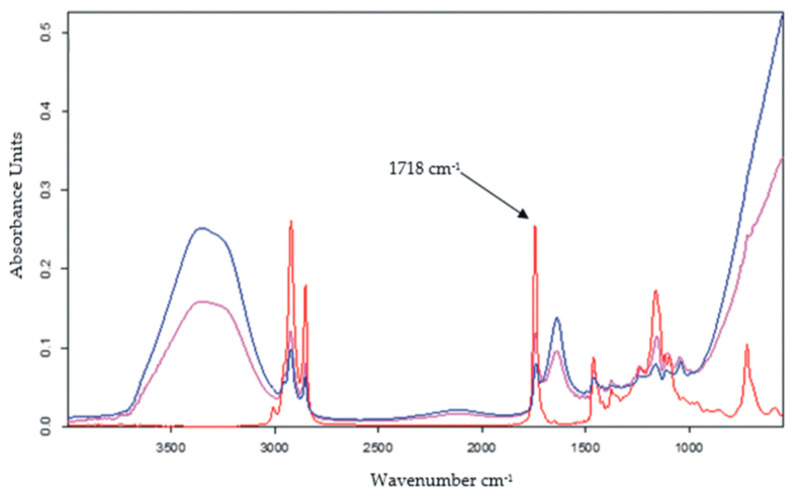
FTIR Spectra for: Apiterra anti-aging cream (blue line), Sabio soothing and repairing balm (red line), and Vivanatura moisturizing mattifying cream (pink line).

FTIR spectra corresponding to commercial products are shown in Figure 15.

### 3.8. Repeatability, Reproducibility and Stability of the GO-Peptide/SPCE

Series of five successive voltammetric measurements using a 10^−3^ M rosmarinic acid solution, each recorded on a newly modified sensor, produced a relative standard deviation of 2.7%. This indicates that GO-Peptide/SPCE provides good reproducibility in the electrochemical determination of rosmarinic acid.

The stability and lifetime of the modified sensor are also important parameters, and hence, these were also analyzed. Therefore, GO-Peptide/SPCE was used for consecutive measurements, without surface renewal, over a period of 30 days, recording its response (anodic peak currents) in a 10^−3^ M rosmarinic acid-0.1 M PBS solution (pH 6.5). The sensor proved to be stable, maintaining 90% of its initial intensity. By keeping the sensor at room temperature and taking measurements every 1–2 days using this solution, no noticeable change in response was evident.

Reproducibility of the manufacturing process was also studied. Three different sensors were modified under identical conditions using the same method, and the responses obtained in a 10^−3^ M rosmarinic acid-0.1 M PBS solution were recorded. The differences between the sensor responses were small, with a relative standard deviation of 2.6%.

## 4. Conclusions

A novel sensor was developed by immobilizing a zwitterionic peptide, containing the H_2_N-D-V-C-Y-Y-A-S-R-COOH sequence, on the surface of an SPE modified with a conductive material with excellent properties, namely a GO composite film. Characterization of both the modified and the unmodified electrode was carried out using voltammetric techniques and FTIR spectroscopy, with the results obtained demonstrating the increased selectivity of the peptide-based sensor for the quantitative determination of rosmarinic acid compared to those obtained with the unmodified electrode. The octapeptide fluorescence quenching studies, performed at pH 6.5, reveal that this bio-inspired molecule interacts slightly differently with electroactive species such as ferricyanide and ferrocyanide ions. The quenching experiments in solution might be performed even by using a 235/302 (ex/em) pair, but with a much lower fluorescence intensity response, as provided by 3D fluorescence quenching data. 

The surface of the modified electrode was characterized by FTIR method and SEM.

The calibration curve of the GO-Peptide/SPCE towards rosmarinic acid showed linearity in the concentration range of 0.1–3.20 µM, with LOD (0.0966 μM) and LOQ (0.322 μM) values close to those obtained by other devices developed for the detection of the same compound, namely rosmarinic acid. Moreover, besides being simple to prepare, the sensor showed good sensitivity, long-term stability, reproducibility, low cost, and acceptable recoveries when tested on real samples, i.e., cosmetic products with different concentrations of rosmarinic acid.

The results obtained in the present study are encouraging for evaluating rosmarinic acid content in different cosmetic products by the voltammetric method. Compared to conventional methods, electrochemical methods have several advantages, including reduced sample analysis time, small sample quantity required, very good accuracy, and portability, favoring the use of this electroanalytical technique for quality control of these products. The method can be extended to the analysis of rosmarinic acid in other types of samples, such as food samples, nutraceutical formulations, or human serum samples.

## Figures and Tables

**Table 1:** Data obtained from the calibration curves for GO-Peptide/SPCE during rosmarinic acid detection.

Electrode	Linear Equation	R^2^	LOD (µmol·L^−1^)	LOQ (µmol·L^−1^)
GO-Peptide/SPCE	Ipa = 0.2951c + 6.6142	0.9984	0.0966	0.322

**Table 2:** Performance characteristics of several electrochemical sensors in the detection of rosmarinic acid.

Sensors	Electroanalytical Technique	LOD (µmol·L^−1^)	Real Samples	References
Biomimetic sensor based on a dinuclear Fe(III)Zn(II) mimetic complex ^1^	SWV	2.3	Plant extracts	[89]
Fe_3_O_4_-Pc-CMWCNTs/MGCE ^2^	DPV	0.182	Leach liquor of plants and human serum samples	[16]
GCE/PoPD/Pt ^3^	DPV	0.50	*Rosmarinus officinalis* L. and *Melissa officinalis* herbs	[8]
BMIBF_4_-CPE and BMIPF_6_-CPE–laccase biosensors ^4^	SWV	0.18	Plant extracts	[21]
GO-Peptide/SPCE	CV	0.09	Cosmetic products	This study

^1^ Fe^3+^Zn^2+^-[bis(2-pyridylmethyl) aminomethyl]-6-[(2-hydroxylbenzyl) (2-pyridyl-methyl) aminomethyl]-4-methyl-phenol; ^2^ Fe_3_O_4_-Pc-CMWCNTs/MGCE-hybrid ferrites composed of Fe and phthalocyanine multiwall carbon nanotubes with magnetic glassy carbon electrode; ^3^ GCE/PoPD/Pt-platinum nanoparticles and poly(ortho-phenylenediamine) film deposited onto a glassy carbon electrode; ^4^ BMIBF_4_-CPE and BMIPF_6_-CPE–laccase biosensors-laccase (1-n-butyl-3-methylimidazolium hexafluorophosphate and 1-nbutyl-3-methylimidazolium tetrafluoroborate) biosensors; SWV—square wave voltammetry; DPV—differential pulse voltammetry.

**Table 3:** Rosmarinic acid concentrations obtained from the two analysis methods used in this study.

Cosmetic Product	c% Rosmarinic Acid Voltammetric Method	c% Rosmarinic Acid FTIR Spectroscopy
Apiterra anti-aging cream	0.288 ± 0.03	0.321 ± 0.05
Sabio soothing and repairing balm	1.042 ± 0.08	1.061 ± 0.09
Vivanatura moisturizing mattifying cream	0.081 ± 0.01	0.076 ± 0.01

## Data Availability

Not applicable.

## References

[1] Neethirajan S., Jayas D.S. (2010). Nanotechnology for the Food and Bioprocessing Industries. Food Bioprocess Technol..

[2] Zielonka J., Joseph J., Sikora A., Hardy M., Ouari O., Vasquez-Vivar J., Cheng G., Lopez M., Kalyanaraman B. (2017). Mitochondria-Targeted Triphenylphosphonium-Based Compounds: Syntheses, Mechanisms of Action, and Therapeutic and Diagnostic Applications. Chem. Rev..

[3] Al-Dhabi N.A., Arasu M.V., Park C.H., Park S.U. (2014). Recent studies on rosmarinic acid and its biological and pharmacological activities. EXCLI J..

[4] Costa D.C., Costa H.S., Albuquerque T.G., Ramos F., Castilho M.C., Sanches-Silva A. (2015). Advances in phenolic compounds analysis of aromatic plants and their potential applications. Trends Food Sci. Technol..

[5] Teixeira J., Gaspar A., Garrido E.M., Garrido J., Borges F. (2013). Hydroxycinnamic Acid Antioxidants: An Electrochemical Overview. BioMed. Res. Int..

[6] Shahidi F., Chandrasekara A. (2009). Hydroxycinnamates and their in vitro and in vivo antioxidant activities. Phytochem. Rev..

[7] Newair E.F., Abdel-Hamid R., Kilmartin P. (2016). Mechanism of Chicoric Acid Electrochemical Oxidation and Identification of Oxidation Products by Liquid Chromatography and Mass Spectrometry. Electroanalysis.

[8] Özdokur K.V., Koçak C. (2019). Simultaneous Determination of Rosmarinic Acid and Protocatechuic Acid at Poly(o-Phenylenediamine)/Pt Nanoparticles Modified Glassy Carbon Electrode. Electroanalysis.

[9] David I.G., Popa D.E., Buleandră M., Cheregi M.C. (2020). Electrochemical Methods and (Bio) Sensors for Rosmarinic Acid Investigation. Chemosensors.

[10] Moreno S., Scheyer T., Romano C.S., Vojnov A.A. (2006). Antioxidant and antimicrobial activities of rosemary extracts linked to their polyphenol composition. Free Radic. Res..

[11] Sotnikova R., Okruhlicova L., Vlkovicova J., Navarova J., Gajdacova B., Pivackova L., Fialova S., Krenek P. (2013). Rosmarinic acid administration attenuates diabetes-induced vascular dysfunction of the rat aorta. J. Pharm. Pharmacol..

[12] Lou K., Yang M., Duan E., Zhao J., Yu C., Zhang R., Zhang L., Zhang M., Xiao Z., Hu W. (2016). Rosmarinic acid stimulates liver regeneration through the mTOR pathway. Phytomedicine.

[13] Nunes S.R.R.P., Madureira A.R., Campos D., Sarmento B., Gomes A.M., Pintado M.M., Reis F. (2015). Therapeutic and Nutraceutical Potential of Rosmarinic Acid-Cytoprotective Properties and Pharmacokinetic Profile. Crit. Rev. Food Sci. Nutr..

[14] Fialová S.B., Kello M., Čoma M., Slobodníková L., Drobná E., Holková I., Garajová M., Mrva M., Zachar V., Lukáč M. (2019). Derivatization of Rosmarinic Acid Enhances its in vitro Antitumor, Antimicrobial and Antiprotozoal Properties. Molecules.

[15] Fonteles A.A., de Souza C.M., Neves J.C.D.S., Menezes A.P.F., Carmo M., Fernandes F.D.P., de Araújo P.R., de Andrade G.M. (2016). Rosmarinic acid prevents against memory deficits in ischemic mice. Behav. Brain Res..

[16] Wang Z., Wang Y., Yang S., Xue L., Feng W., Liu X., Li B., Yin M., Jiao J., Chen Q. (2021). Electrochemical sensor based on magnetic nanohybrids of multiple phthalocyanine doped ferrites/CMWCNTs for detection of rosmarinic acid. Talanta.

[17] Ozturk N., Tunçel M., Uysal D., Oncu-Kaya E.M., Koyuncu O. (2010). Determination of Rosmarinic Acid by High-Performance Liquid Chromatography and Its Application to Certain Salvia Species and Rosemary. Food Anal. Methods.

[18] Casanova F., Estevinho B., Santos L. (2016). Preliminary studies of rosmarinic acid microencapsulation with chitosan and modified chitosan for topical delivery. Powder Technol..

[19] Fachel F.N.S., Nemitz M.C., Medeiros-Neves B., Veras K.S., Bassani V.L., Koester L.S., Henriques A.T., Teixeira H.F. (2018). A novel, simplified and stability-indicating high-throughput ultra-fast liquid chromatography method for the determination of rosmarinic acid in nanoemulsions, porcine skin and nasal mucosa. J. Chromatogr. B.

[20] Saltas D., Pappas C.S., Daferera D., Tarantilis P.A., Polissiou M.G. (2013). Direct Determination of Rosmarinic Acid in *Lamiaceae* Herbs Using Diffuse Reflectance Infrared Fourier Transform Spectroscopy (DRIFTS) and Chemometrics. J. Agric. Food Chem..

[21] Franzoi A.C., Dupont J., Spinelli A., Vieira I.C. (2009). Biosensor based on laccase and an ionic liquid for determination of rosmarinic acid in plant extracts. Talanta.

[22] Munteanu I.G., Apetrei C. (2021). Analytical Methods Used in Determining Antioxidant Activity: A Review. Int. J. Mol. Sci..

[23] Brondani D., Zapp E., Vieira I.C., Dupont J., Scheeren C.W. (2011). Gold nanoparticles in an ionic liquid phase supported in a biopolymeric matrix applied in the development of a rosmarinic acid biosensor. Analyst.

[24] Diaconu M., Litescu S.C., Radu G.L. (2010). Bienzymatic sensor based on the use of redox enzymes and chitosan–MWCNT nanocomposite. Evaluation of total phenolic content in plant extracts. Mikrochim. Acta.

[25] Eremia S.A., Vasilescu I., Radoi A., Litescu S.-C., Radu G.-L. (2013). Disposable biosensor based on platinum nanoparticles-reduced graphene oxide-laccase biocomposite for the determination of total polyphenolic content. Talanta.

[26] Srivastava S., Ali A., Umrao S., Parashar U.K., Srivastava A., Sumana G., Malhotra B.D., Pandey S.S., Hayase S. (2014). Graphene Oxide-Based Biosensor for Food Toxin Detection. Appl. Biochem. Biotechnol..

[27] Sun Y., Wang B., Deng Y., Cheng H., Li X., Yan L., Li G., Sun W. (2021). Reduced graphene oxide/titanium carbide MXene nanocomposite-modified electrode for electrochemical hemoglobin biosensor. J. Chin. Chem. Soc..

[28] Chiticaru E.A., Pilan L., Damian C.-M., Vasile E., Burns J.S., Ioniţă M. (2019). Influence of Graphene Oxide Concentration when Fabricating an Electrochemical Biosensor for DNA Detection. Biosensors.

[29] Santiago E., Poudyal S.S., Shin S.Y., Yoon H.J. (2022). Graphene Oxide Functionalized Biosensor for Detection of Stress-Related Biomarkers. Sensors.

[30] Goyal D., Mittal S.K., Choudhary A., Dang R.K. (2021). Graphene: A two dimensional super material for sensor applications. Mater. Today Proc..

[31] Dikin D.A., Stankovich S., Zimney E.J., Piner R.D., Dommett G.H.B., Evmenenko G., Nguyen S.T., Ruoff R.S. (2007). Preparation and characterization of graphene oxide paper. Nature.

[32] Razaq A., Bibi F., Zheng X., Papadakis R., Jafri S.H.M., Li H. (2022). Review on Graphene-, Graphene Oxide-, Reduced Graphene Oxide-Based Flexible Composites: From Fabrication to Applications. Materials.

[33] Jamaluddin R.Z.A.R., Tan L.L., Chong K.F., Heng L.Y. (2020). An electrochemical DNA biosensor fabricated from graphene decorated with graphitic nanospheres. Nanotechnology.

[34] Sun X., Liu Z., Welsher K., Robinson J.T., Goodwin A., Zaric S., Dai H. (2008). Nano-graphene oxide for cellular imaging and drug delivery. Nano Res..

[35] Munteanu I.G., Apetrei C. (2022). A Review on Electrochemical Sensors and Biosensors Used in Assessing Antioxidant Activity. Antioxidants.

[36] Zhang Y., Wu C., Guo S., Zhang J. (2013). Interactions of graphene and graphene oxide with proteins and peptides. Nanotechnol. Rev..

[37] Fan X., Deng D., Chen Z., Qi J., Li Y., Han B., Huan K., Luo L. (2021). A sensitive amperometric immunosensor for the detection of carcinoembryonic antigen using ZnMn2O4@reduced graphene oxide composites as signal amplifier. Sens. Actuators B Chem..

[38] Li Y., Zhang Z., Zhang Y., Deng D., Luo L., Han B., Fan C. (2016). Nitidine chloride-assisted bio-functionalization of reduced graphene oxide by bovine serum albumin for impedimetric immunosensing. Biosens. Bioelectron..

[39] Mascini M., Palchetti I., Tombelli S. (2011). Nucleic Acid and Peptide Aptamers: Fundamentals and Bioanalytical Aspects. Angew. Chem. Int. Ed..

[40] Sfragano P., Moro G., Polo F., Palchetti I. (2021). The Role of Peptides in the Design of Electrochemical Biosensors for Clinical Diagnostics. Biosensors.

[41] Puiu M., Bala C. (2018). Peptide-based biosensors: From self-assembled interfaces to molecular probes in electrochemical assays. Bioelectrochemistry.

[42] Tertis M., Hosu O., Feier B., Cernat A., Florea A., Cristea C. (2021). Electrochemical Peptide-Based Sensors for Foodborne Pathogens Detection. Molecules.

[43] Zhang Q., Zhang D., Lu Y., Yao Y., Li S., Liu Q. (2015). Graphene oxide-based optical biosensor functionalized with peptides for explosive detection. Biosens. Bioelectron..

[44] Ye H., Wang L., Huang R., Su R., Liu B., Qi W., He Z. (2015). Superior Antifouling Performance of a Zwitterionic Peptide Compared to an Amphiphilic, Non-Ionic Peptide. ACS Appl. Mater. Interfaces.

[45] Wang G., Han R., Su X., Li Y., Xu G., Luo X. (2017). Zwitterionic peptide anchored to conducting polymer PEDOT for the development of antifouling and ultrasensitive electrochemical DNA sensor. Biosens. Bioelectron..

[46] Chernousova S., Epple M. (2013). Silver as Antibacterial Agent: Ion, Nanoparticle, and Metal. Angew. Chem. Int. Ed..

[47] Tischer M., Pradel G., Ohlsen K., Holzgrabe U. (2011). Quaternary Ammonium Salts and Their Antimicrobial Potential: Targets or Nonspecific Interactions?. ChemMedChem.

[48] Zhang P., Lin L., Zang D., Guo X., Liu M. (2016). Designing Bioinspired Anti-Biofouling Surfaces based on a Superwettability Strategy. Small.

[49] Shao Q., Jiang S. (2014). Molecular Understanding and Design of Zwitterionic Materials. Adv. Mater..

[50] Sun Q., Yan F., Yao L., Su B. (2016). Anti-Biofouling Isoporous Silica-Micelle Membrane Enabling Drug Detection in Human Whole Blood. Anal. Chem..

[51] Lowe S., O’Brien-Simpson N.M., Connal L.A. (2014). Antibiofouling polymer interfaces: Poly(ethylene glycol) and other promising candidates. Polym. Chem..

[52] Wang G., Su X., Xu Q., Xu G., Lin J., Luo X. (2018). Antifouling aptasensor for the detection of adenosine triphosphate in biological media based on mixed self-assembled aptamer and zwitterionic peptide. Biosens. Bioelectron..

[53] Munteanu I.G., Apetrei C. (2022). Tyrosinase-Based Biosensor—A New Tool for Chlorogenic Acid Detection in Nutraceutical Formulations. Materials.

[54] Munteanu I.-G., Apetrei C. (2021). Electrochemical Determination of Chlorogenic Acid in Nutraceuticals Using Voltammetric Sensors Based on Screen-Printed Carbon Electrode Modified with Graphene and Gold Nanoparticles. Int. J. Mol. Sci..

[55] Li Y., Deng D., Wang H., Huan K., Yan X., Luo L. (2021). Controlled synthesis of Cu-Sn alloy nanosheet arrays on carbon fiber paper for self-supported nonenzymatic glucose sensing. Anal. Chim. Acta.

[56] Munteanu I.G., Apetrei C. (2021). A Review on Electrochemical Sensors and Biosensors Used in Chlorogenic Acid Electroanalysis. Int. J. Mol. Sci..

[57] Ma M., Fan X.P., Dai Z., Liu X., Xu S.C., Wei J., Shi S., Chen G.P. (2012). Graphene Oxide Modified DNA Electrochemical Biosensors. Appl. Mech. Mater..

[58] Liao L., Pan C. (2011). Enhanced Electrochemical Capacitance of Nitrogen-Doped Carbon Nanotubes Synthesized from Amine Flames. Soft Nanosci. Lett..

[59] Chen J., Chen Q., Ma Q. (2012). Influence of surface functionalization via chemical oxidation on the properties of carbon nanotubes. J. Colloid Interface Sci..

[60] He Q., Sudibya H.G., Yin Z., Wu S., Li H., Boey F., Huang W., Chen P., Zhang H. (2010). Centimeter-Long and Large-Scale Micropatterns of Reduced Graphene Oxide Films: Fabrication and Sensing Applications. ACS Nano.

[61] Danielsen S.P.O., Beech H.K., Wang S., El-Zaatari B.M., Wang X., Sapir L., Ouchi T., Wang Z., Johnson P.N., Hu Y. (2021). Molecular Characterization of Polymer Networks. Chem. Rev..

[62] Liu J., Fu S., Yuan B., Li Y., Deng Z. (2010). Toward a Universal “Adhesive Nanosheet” for the Assembly of Multiple Nanoparticles Based on a Protein-Induced Reduction/Decoration of Graphene Oxide. J. Am. Chem. Soc..

[63] Bagińska K., Makowska J., Wiczk W., Kasprzykowski F., ChmurzyńSKI L. (2007). Conformational studies of alanine-rich peptide using CD and FTIR spectroscopy. J. Pept. Sci..

[64] Goormaghtigh E., Ruysschaert J.-M., Raussens V. (2006). Evaluation of the Information Content in Infrared Spectra for Protein Secondary Structure Determination. Biophys. J..

[65] Surewicz W.K., Mantsch H.H., Chapman D. (1993). Determination of protein secondary structure by Fourier transform infrared spectroscopy: A critical assessment. Biochemistry.

[66] Zhang H., Lv X., Li Y., Wang Y., Li J. (2009). P25-Graphene Composite as a High Performance Photocatalyst. ACS Nano.

[67] Miron-Mendoza M., Graham E., Manohar S., Petroll W.M. (2017). Fibroblast-fibronectin patterning and network formation in 3D fibrin matrices. Matrix Biol..

[68] Kivanany P.B., Grose K.C., Yonet-Tanyeri N., Manohar S., Sunkara Y., Lam K.H., Schmidtke D.W., Varner V.D., Petroll W.M. (2018). An In Vitro Model for Assessing Corneal Keratocyte Spreading and Migration on Aligned Fibrillar Collagen. J. Funct. Biomater..

[69] Saeidi N., Sander E.A., Ruberti J.W. (2009). Dynamic shear-influenced collagen self-assembly. Biomaterials.

[70] Konda M., Bhowmik S., Mobin S.M., Biswas S., Das A.K. (2016). Modulating Hydrogen Bonded Self-assembled Patterns and Morphological Features by a Change in Side Chain of Third Amino Acid of Synthetic γ-Amino Acid Based Tripeptides. ChemistrySelect.

[71] Amina M., Al Musayeib N.M., Alarfaj N.A., El-Tohamy M.F., Al-Hamoud G.A., Alqenaei M.K.M. (2022). The Fluorescence Detection of Phenolic Compounds in *Plicosepalus curviflorus* Extract Using Biosynthesized ZnO Nanoparticles and Their Biomedical Potential. Plants.

[72] Yang J., Kim S.-E., Cho M., Yoo I.-K., Choe W.-S., Lee Y. (2014). Highly sensitive and selective determination of bisphenol—A using peptide-modified gold electrode. Biosens. Bioelectron..

[73] Bounegru A.V., Apetrei C. (2020). Development of a Novel Electrochemical Biosensor Based on Carbon Nanofibers–Gold Nanoparticles–Tyrosinase for the Detection of Ferulic Acid in Cosmetics. Sensors.

[74] Bounegru A.V., Apetrei C. (2021). Development of a Novel Electrochemical Biosensor Based on Carbon Nanofibers–Cobalt Phthalocyanine–Laccase for the Detection of p-Coumaric Acid in Phytoproducts. Int. J. Mol. Sci..

[75] Munteanu I.G., Apetrei C. (2022). Assessment of the Antioxidant Activity of Catechin in Nutraceuticals: Comparison between a Newly Developed Electrochemical Method and Spectrophotometric Methods. Int. J. Mol. Sci..

[76] Abrha T., Pal R., Saini R.C. (2017). A Study on Voltametric Electro-kinetic Mechanism of Catechol at l-glutamic Acid-Carbon Paste Sensor. J. Surf. Sci. Technol..

[77] Shamsi A., Shahwan M., Husain F.M., Khan M.S. (2019). Characterization of methylglyoxal induced advanced glycation end products and aggregates of human transferrin: Biophysical and microscopic insight. Int. J. Biol. Macromol..

[78] Miura T., Suzuki K., Takeuchi H. (2001). Binding of iron (III) to the single tyrosine residue of amyloid β-peptide probed by Raman spectroscopy. J. Mol. Struct..

[79] Petrucci R., Bortolami M., Di Matteo P., Curulli A. (2022). Gold Nanomaterials-Based Electrochemical Sensors and Biosensors for Phenolic Antioxidants Detection: Recent Advances. Nanomaterials.

[80] Curulli A. (2020). Nanomaterials in Electrochemical Sensing Area: Applications and Challenges in Food Analysis. Molecules.

[81] Xiong Y.L., Mine Y., Li-Chan E., Jiang B. (2010). Antioxidant Peptides. Bioactive Proteins and Peptides as Functional Foods and Nutraceuticals.

[82] Cornejo A., Sandoval F.A., Caballero L., Machuca L., Muñoz P., Caballero J., Perry G., Ardiles A., Areche C., Melo F. (2017). Rosmarinic acid prevents fibrillization and diminishes vibrational modes associated to β sheet in tau protein linked to Alzheimer’s disease. J. Enzym. Inhib. Med. Chem..

[83] Papaemmanouil C., Chatziathanasiadou M.V., Chatzigiannis C., Chontzopoulou E., Mavromoustakos T., Grdadolnik S.G., Tzakos A.G. (2020). Unveiling the interaction profile of rosmarinic acid and its bioactive substructures with serum albumin. J. Enzym. Inhib. Med. Chem..

[84] Gil E., Enache T., Oliveira-Brett A. (2013). Redox Behaviour of Verbascoside and Rosmarinic Acid. Comb. Chem. High Throughput Screen..

[85] Bilgi M., Sahin E.M., Ayranci E. (2018). Sensor and biosensor application of a new redox mediator: Rosmarinic acid modified screen-printed carbon electrode for electrochemical determination of NADH and ethanol. J. Electroanal. Chem..

[86] Mohamadi M., Mostafavi A., Torkzadeh-Mahani M. (2015). Voltammetric Determination of Rosmarinic Acid on Chitosan/Carbon Nanotube Composite-Modified Carbon Paste Electrode Covered with DNA. J. Electrochem. Soc..

[87] Bounegru A.V., Apetrei C. (2022). Simultaneous Determination of Caffeic Acid and Ferulic Acid Using a Carbon Nanofiber-Based Screen-Printed Sensor. Sensors.

[88] Apetrei I.M., Apetrei C. (2016). Amperometric Biosensor Based on Diamine Oxidase/Platinum Nanoparticles/Graphene/Chitosan Modified Screen-Printed Carbon Electrode for Histamine Detection. Sensors.

[89] Santhiago M., Peralta R.A., Neves A., Micke G.A., Vieira I.C. (2008). Rosmarinic acid determination using biomimetic sensor based on purple acid phosphatase mimetic. Anal. Chim. Acta.

[90] Fahelelbom K.M., Saleh A., Al-Tabakha M.M., Ashames A.A. (2022). Ashames, Recent applications of quantitative analytical FTIR spectroscopy in pharmaceutical, biomedical, and clinical fields: A brief review. Rev. Anal. Chem..

[91] Tawfeeq A.A., Faisal M., Abaas I., Alwan A. (2018). Isolation, quantification, and identification of rosmarinic acid, gas chromatography-mass spectrometry analysis of essential oil, cytotoxic effect, and antimicrobial investigation of rosmarinus officinalis leaves. Asian J. Pharm. Clin. Res..

